# Older Worker-Orientated Human Resource Practices, Wellbeing and Leave Intentions: A Conservation of Resources Approach for Ageing Workforces

**DOI:** 10.3390/ijerph20032725

**Published:** 2023-02-03

**Authors:** Ben Farr-Wharton, Tim Bentley, Leigh-ann Onnis, Carlo Caponecchia, Abilio De Almeida Neto, Sharron O’Neill, Catherine Andrew

**Affiliations:** 1 School of Business and Law, Edith Cowan University, Perth, WA 6027, Australia; 2 Centre for Work + Wellbeing, Edith Cowan University, Perth, WA 6027, Australia; 3 College of Business, Law & Governance, James Cook University, Cairns, QLD 4870, Australia; 4 School of Aviation, University of NSW, Sydney, NSW 2052, Australia; 5 Centre for Work Health and Safety, NSW Government, Sydney, NSW 2000, Australia; 6 School of Business, University of NSW, Canberra, ACT 2612, Australia; 7 School of Health and Society, University of Wollongong, Wollongong, NSW 2500, Australia

**Keywords:** mature-age human resource practices, older workers, mental health, turnover intentions, age-discrimination, ageism, work-family conflict

## Abstract

At a time where there are ageing populations, global shortages of skilled labour, and migration pathways impacted by the COVID-19 pandemic, retaining older workers presents as a vital strategic initiative for organizations globally. This study examines the role of Human Resource Practices (HRPs), which are oriented towards accommodating the needs of an ageing workforce in mitigating psychological distress and turnover intentions. The study collected self-reported survey data from 300 Australian employees over the age of 45, over two time points. Using structural equation modelling, the study analyzed the extent to which Older Worker-oriented Human Resources Practices (OW-HRPs) translate into employee psychological health and retention within organizations, through the mediation of ageism and work–life conflict. The results support our hypothesis that OW-HRPs are associated with lower ageism, better work–life balance; and in combination these reduce psychological distress and help retain older workers in the workforce. We conclude that OW-HRPs can foster work environments conducive to older worker wellbeing, supporting the retention of talent and maintaining effectiveness, in the face of substantial labour supply challenges brought on by the COVID-19 pandemic and an ageing population.

## 1. Introduction

The rate of workforce participation for older workers is increasing. With global ageing population growth, as well as a multitude of factors, most notably improved general health, voluntary retirement ages, access to superannuation, and increasingly insecure employment, workers are remaining in the workforce for longer [1,2,3,4]. Maintaining and growing workforce participation rates of older workers is vitally important [5], particularly as the COVID-19 pandemic stemmed the migration of skilled workers, thereby further increasing pressures on existing and ageing workforces. Increased workforce participation, together with an impetus to retain older workers at a time when migration pathways are impacted by the COVID-19 pandemic, presents as a vital strategic yet challenging concern for organizations.

In Australia, the population is ageing rapidly, with the Australian Institute of Health and Welfare [6] reporting that workforce participation rates for workers aged 65 and over has more than doubled in the last twenty years. Along with increasing participation, there is growing awareness concerning the impact of ‘ageism’ (i.e., stereotypes, or how we think about ageing) [7] and ‘age discrimination’ (i.e., actions, or how we act towards others based on age) [7], in the Australian workforce [8,9,10] and internationally [5,11]. In 2021, the Australian Human Rights Commission (AHRC) released their report entitled, ‘What’s age got to do with it?’, highlighting that 63% of people had reported experiencing ageism in the past five years. Alarmingly, those aged between 40–61 experienced ageism in the form of exclusion from further employment or promotion [12]. With an increasing prevalence of ageing workforces and the critical need to engage and retain older workers within many industries, organizations need human resource practices (HRPs) designed to meet the specific needs of older workers and enhance their wellbeing and longevity in employment.

### 1.1. Wellbeing, Age and the Work Environment

The current literature suggests that it is the organisation of work and the work environment, rather than chronological age per se, that influences older workers’ wellbeing and, therefore, the length of their working life [1,13,14]. Further, in their rapid review of the impact of COVID-19 on the labour market, work, and workers, Bellotti et al. [15] found differences in levels of turnover intention for older and younger workers across various job types worldwide. Increasingly, work environments fostering a psychological safety climate [9] and a positive age-diversity climate [1] influence the work experience for older workers and their turnover intention. Therefore, the drive to improve retention of older workers comes against a backdrop of increasing awareness concerning age discrimination and/or ageism at work [1,9]. Hence, it appears that many organisations are grappling with the dual dynamic of retaining older workers while simultaneously changing retrograde cultural norms that exclude workers on the basis of age.

### 1.2. HRM Practices and Managing Older Workers

A growing body of evidence suggests that when managing older workers, a specific set of HR practices are required [10,16]. The Older Worker-oriented Human Resource Practices (OW-HRPs) (sometimes referred to as HR mature-age practices) comprise a set of policies and processes that organizations can put in place to offer recognition and support, thus catering for the changing needs of staff over the age of 45 years [13,17,18,19]. These distinctive HR practices for managing older workers provide for differences in the motivations and values typically seen in younger and older workers [9]. Building on the work of Kulik et al. [10] and Teo et al. [9], OW-HRPs comprise practices which alleviate age discrimination and focus on continued meaningful work opportunities and career paths for older workers. These include mentorship, training, rewards and incentives, performance management, flexible working conditions and graduated retirement pathways. More specifically, OW-HRPs may involve creating new roles or redesigning work so it is appealing to older employees; providing meaningful work that meets the needs of the organization (e.g., productivity) and the older worker (e.g., work schedule); providing flexible work arrangements (e.g., job sharing, work-from-home, reduced work hours); providing older workers with an opportunity to have input into workload allocation; and supporting older workers to access training for skill development (including assistance with the acquisition of skills to use new technology).

OW-HRPs are considered organizational resources and are typical of those found to influence the health, safety and wellbeing outcomes for older workers [9]. For example, several studies have found benefits with flexible working (e.g., part-time work, shift scheduling) which have been linked to positive health and wellbeing outcomes for older workers [20,21,22,23,24]. Further, there is a growing body of evidence that OW-HRPs are positively associated with retention, wellbeing [5,9,13,17,18,19], and workability amongst older workers with chronic health conditions [25]. Furthermore, Teo et al. [9] found that HR mature-age practices were associated with improved wellbeing and that ‘mature-age HR practices positively and partially mediated the relationship between inclusive leadership and psychological wellbeing’ (p. 323). Given the contemporary workplace challenges associated with an ageing population and the predicted skill shortages across many professions, understanding the OW-HRPs that support older workers’ continued workforce participation is increasingly important [5,26].

Where there is an imperative to do more to retain older workers, one approach is through OW-HRPs that support healthy transitions towards retirement (e.g., graduated retirement, flexible working), promote healthy ageing at work, and strengthen psychological health through eliminating ageism and fostering inclusive organisational cultures. As such, OW-HRPs have a role in mitigating ageism, retaining talent and managing the successful transition of younger workers into, and older workers out of, organisations.

### 1.3. Study Aim

Our study examined the role of HRPs oriented towards accommodating the needs of an ageing workforce. To add to the current evidence, the overarching aim was to examine the role of OW-HRPs in mitigating psychological distress and subsequent turnover intentions using the Conservation of Resources [27] theory.

### 1.4. Theory

This study used Conservation of Resources (COR) theory [27], which proposes that ‘individuals strive to obtain, retain, foster, and protect those things they value’ to reduce their experience of stress due to resource loss (p. 104). Of interest to this study about older workers is the *Primacy of loss principle*, whereby ‘resource loss is disproportionately more salient than resource gain’, and the *Gain paradox principle* whereby at times of great resource loss, resource gains become more important [27] (p. 106). As such, COR theory provides insight into the role of HRPs for older workers, such as: investing in resources for older workers; supporting older workers to mitigate the speed with which they are experiencing resource loss; and providing access to the supports needed to acquire and accumulate the resources older workers need – in other words, improving access to resources to reduce the impact of stress and negative health outcomes to improve the retention of experienced, skilled older workers. One area of critical resources with the potential to positively influence the work environment and how older workers experience work is HRPs. Therefore, COR theory provides a lens through which to examine the role of OW-HRPs in mitigating psychological distress and turnover intentions.

Interest in understanding how HRPs contribute to the retention of older workers has increased over the past decade [9,10,17,18,28]. Kulik et al. [10], for example, argue for the implementation of HR ‘mature-age’ practices with the goal of reducing exposure to age-related stereotype threats faced by older workers impacting their engagement with work. While HRM scholars, led by Guest [29], have argued that HRM should be central to the psychological wellbeing of employees, research seeking to link HRPs and the psychological wellbeing of older workers is in its infancy [30]. Early evidence suggests that older workers experience positive wellbeing when they are provided with opportunities for skill development and recognition for their work contributions and achievements [13,19,30].

### 1.5. Hypotheses

The following section describes the hypothesis development for the relationships between OW-HRPs, Psychological Distress, Intention to Leave, Work–life Conflict and Ageism. The literature supporting the development of the five hypotheses is discussed with the hypothetic model presented at the end of this section.

#### 1.5.1. OW-HRPs and Leave Intention

OW-HRPs include multiple measures to support retention and the productive work engagement of older workers. The influence of OW-HRPs on leave intention is increasing associated with the work-related (e.g., career paths, access to training, job security) and social ties (e.g., belongingness, respect) that reduce turnover intention for older workers [9,10,26,31]. In a study of post-retirement employees, Armstrong-Stassen [17] found that HR practices, such as ‘providing flexible work schedules (days worked, hours worked)’ and part-time work schedules were ‘important in influencing their decision to remain in the workforce’ (p. 43–44). Moreover, part-time work was found to be a strong predictor of good health and wellbeing for older workers in an Australian [21] and Japanese study [22]. Similarly, Bentley et al. [11] found an association between OW-HRPs that included organisational support (including protection from age discrimination) and leave intention. Consistent with previous research in the field [10,11,16], our study examined OW-HRPs which focus on respecting, valuing and recognizing the contributions of older workers, and the design of work to accommodate the needs, preferences and limitations of older workers.

Using a COR lens, OW-HRPs play an important role by signalling that the organization cares about the wellbeing and psychological health of older workers [10], practices that can reduce job demands and associated job stress by offsetting resource loss and enhancing feelings of belongingness, purpose and value to the organization [16]. These enhanced feelings of being supported, and their impact on the wellbeing and commitment to remain working for the organization, can be understood through COR’s *Resource investment principle*, which states that individuals invest in resources for three reasons—to gain resources, to recover from a loss of resources, or to protect against future resource loss [27]. As such, an investment in resources is beneficial to both the older worker and the organization. Therefore, we posit that:

**Hypothesis** **1 (H1).** *OW-HRPs are negatively associated with turnover intention.*

#### 1.5.2. Psychological Distress

OW-HRPs provide a critical organizational resource that can help offset resource loss and associated stress [27] and psychological distress (i.e., reduce anxiety and depression related to employment conditions (e.g., job security) and/or the work environment (e.g., ageism) for older workers) [10,20,31]. Using common experiences of older workers as examples, COR theory proposes that stress occurs when an individual is threatened with loss (e.g., ageism, forced retirement), experiences actual loss (e.g., overlooked for promotion), or fails to gain key resources (access to professional development); however this is mitigated by the social and material resources that an individual has available to them (acquired and conserved) at the time (e.g., health, well-being, family, and purpose) [27]. Given that psychological distress is associated with negative work experiences, we posit that:

**Hypothesis** **2 (H2).** *Psychological distress mediates the relationship linking OW-HRPs with Turnover Intention.*

#### 1.5.3. Work–life Conflict

Work–life conflict occurs when the demands of a person’s work role conflicts with those of their family role, and where the domains of work and non-work life are mutually incompatible in some way [32,33]. Higher levels of work–life conflict are considered a resource loss, predicting stress in older workers where this is unmanaged. Indeed, extant research indicates that work–life conflict is negatively associated with organizational commitment and positively associated with turnover intention and poor health outcomes [20,34]. Few studies have specifically considered work–life conflict amongst older workers [33,35] and its impact on health and retirement. Notable studies include a meta-analysis by Allen et al. [36] finding an association with health outcomes and retirement intentions and a study by Garcia et al. [34] also finding work–life conflict to be associated with retirement intentions.

Hence, the available research suggests that work–life conflict impacts older workers and appears to predict early retirement [34,37] and/or leaving the profession [20], suggesting that it influences turnover intentions. Several studies found that HRPs can be effective in reducing work–life conflict, which in turn can promote employee wellbeing and retention [19,38,39]. Therefore, based on evidence for the role of work–life conflict on the wellbeing and retention outcomes of the wider workforce, and limited evidence for these impacts on older workers, we posit that:

**Hypothesis** **3 (H3).** *OW-HRPs are negatively associated with Work–Life Conflict.*

#### 1.5.4. Ageism

Exposure to ageism and age discrimination has a negative impact on the work environment, acts as a stressor [40], and is often experienced by older workers [5,8,9,10,11]. Age discrimination can result in poor mental health and turnover intentions as workers perceive a sense of being devalued, disrespected and psychologically unsafe at work [10,11,26]. Age discrimination can negatively impact various aspects of an employee’s work–life, including recruitment (i.e., access to employment), retirement decisions, promotions, training, remuneration and exclusion from roles involving new technology [31,41]. While there is an absence of research that has specifically examined the structural relationship between OW-HRPs and age discrimination, using the COR theory it is proposed that OW-HRPs will help older workers balance the demands in their work environment. We, therefore, posit:

**Hypothesis** **4 (H4).** *OW-HRPs are negatively associated with Age discrimination.*

#### 1.5.5. Ageism and Work–Life Conflict

As discussed earlier, OW-HRPs operate as a form of organizational resource that can help offset resource loss and increased demands associated with exposure to ageism for older workers, thereby reducing the experience of stress [27]. At the same time, ageism has been associated with unwanted outcomes, including early retirement [15,42], turnover intention [26] and employee mental health problems [9,11,43]. Discriminatory practices represent a significant resource loss for older workers where unmanaged and can impact work–life balance negatively. We, therefore, posit:

**Hypothesis** **5 (H5).** *Ageism, Work–Life Conflict mediate the relationship linking OW-HRPs with Turnover Intention.*

## 2. Methods

### 2.1. Study Design

This quantitative study surveyed older workers, over two time points during the COVID-19 pandemic. The data were analyzed using structural equation modelling in SPSS (Statistical Package for the Social Sciences) and the Analysis of Moment Structures (AMOS) plugin (version 28).

### 2.2. Defining Older Workers

Currently, there is no universal definition, or classification, as to when someone is an ‘older worker’. Therefore, this study uses an accepted approach whereby older workers are classed as those aged 45 and older, which is consistent with the definition of the Australian Bureau of Statistics [44] and research in this field [5,8].

### 2.3. Participants and Sample Size

The non-identifiable, self-report survey data was collected from a panel sample of Australian employees aged 45 years and over, at two time points. A total of 1026 older workers responded in time one (September 2021). Of that original sample, 300 older workers responded in time two (two weeks after time one). There was an attrition rate of 71%. Table 1 shows the characteristics of the participants in our study.

### 2.4. Instruments

#### 2.4.1. OW-HRPs

OW-HRPs were measured using a 14-item scale adapted from Armstrong-Stassen [17]. An example item includes, ‘to what extent is your organization currently engaging in each of the following human resource practices?’—‘redesigning jobs to be more appealing to mature employees’; ‘providing a reduced work week’. Using a five-point Likert scale, values ranged from 1 (not doing this at all) to 5 (highly engaged in doing this).

#### 2.4.2. Turnover Intention

Turnover Intention was measured using a three-item scale. An example item includes, ‘It is likely that I would actually leave my current organization within the next year.’ Using a five-point Likert scale, values ranged from 1 (strongly disagree) to 5 (strongly agree).

#### 2.4.3. Psychological Distress

Psychological distress was measured using the Kessler 6 Psychological Distress Scale (K6+). An example item includes, ‘during the past 30 days, how often did you feel hopeless?’. A five-point Likert scale was used, where ‘1’ corresponded with ‘none of the time’ and ‘5’ corresponded with ‘all of the time’.

#### 2.4.4. Work–life Conflict

Work–life conflict was measured using the five-item scale provided by the Copenhagen Psychological Questionnaire (COPSOQ III). An example item includes, ‘my work drains so much of my energy that it has a negative effect on my private life.’ Using a five-point Likert scale, values ranged from 1 (strongly disagree) to 5 (strongly agree).

#### 2.4.5. Ageism

Ageism was measured using the nine-item ‘attitudes and behaviours towards older workers’ scale. An example item includes, ‘In my organization, there is age-discriminatory behaviour regarding opportunities for individual promotion.’ Using a five-point Likert scale, values ranged from 1 (strongly disagree) to 5 (strongly agree).

### 2.5. Hypothetical Model

**Figure 1:**
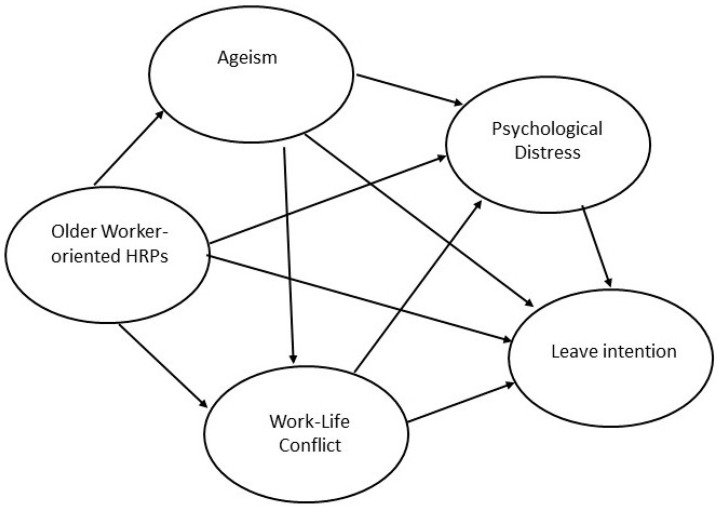
Hypothetical model.

Figure 1 shows the hypothetical model employed for this study, incorporating the hypotheses.

### 2.6. Construct Reliability and Validity

A Confirmatory Factor Analysis model was constructed in the Analysis of Moment Structures (AMOS) program, so as to explore instrument reliability, discriminant and convergent validity. The initial model fit for the CFA was adequate, though not ideal, with a corrected fit index (CFI) of 0.906; a Tucker Lewis Index (TLI) of 0.899; a chi-squared over degrees (x^2^/df) of freedom metric of 2.191, and a Root Mean Square Estimation Error of Approximation (RMSEA) of 0.063. The analysis indicated that the model would benefit from several modifications. As such, covariances were drawn between the first and third items of K6+; the first and fourth, and the second and third items of ageism. Several items of the OW-HRPs sub-scales were deleted to strengthen parsimony, leaving each of the subconstructs identified by four items. The modifications improved the model fit, with the new CFI and TLI being 0.933 and 0.927, respectively; the x^2^/df equality being 1.857; and the RMSEA being 0.054. Table 2 below highlights the instrument reliability and validity indices, including the inter-construct correlations. All constructs presented as statistically reliable and sufficiently discriminant from each other, as displayed in Table 2. Accordingly, a path model was assembled to test the hypotheses.

## 3. Results

Descriptive and correlation statistics of the modelled constructs are displayed in Table 3.

The fit indices for the final path were appropriate, with a CFI and TLI being 0.994 and 0.968, respectively; the x^2^/df being 1.774; and the RMSEA being 0.051. Mediation testing used bias-corrected bootstrapped confidence interval assessment, bootstrapped to 4000 samples. Table 4 shows the direct and indirect effects of the modelled paths.

**Figure 2:**
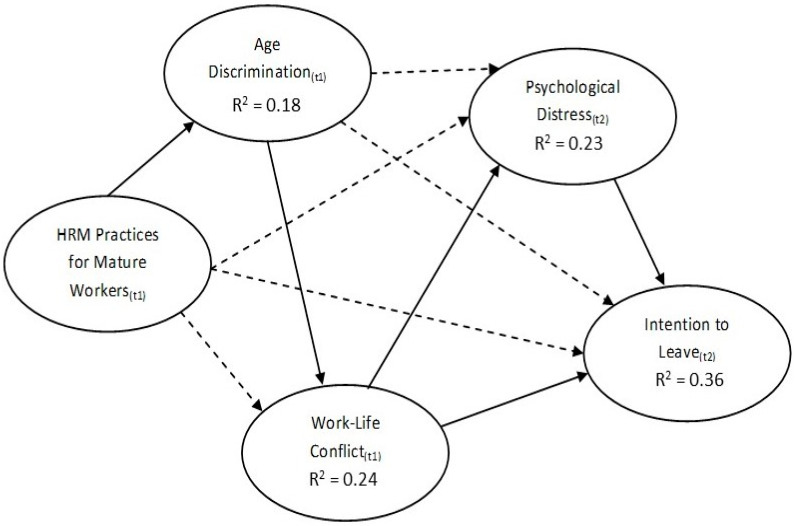
Path model R^2^ results. The complete line = significant path; the dotted line = mediation pathway; t1 = the data obtained from the first wave of collection (time 1); and t2 = data obtained from the second wave of collection (time 2).

The path analysis indicated support for all tested hypotheses (Figure 2). The modelled variables accounted for 36% of the variance of turnover intention, indicating a low to moderate predictive weight. While not significant, the control variable age cohort (45–54, 55–64, 65+) had a *p* value below 0.1 but above 0.05 (which is sometimes referred to as ‘approaching significance’); which indicates a slight but not significant trend where those in the younger age brackets had lower turnover intention scores.

Our study developed a chain mediation model to empirically test the effect of OW-HRPs on psychological distress and intention to quit in a sample of older workers. We found support for each of our hypotheses. The direct effects of OW-HRPs on older worker wellbeing and leave intentions were mediated by work–life conflict, while age-discrimination further weakened the potency of OW-HRPs to impact positive outcomes by fully mediating the relationship between OW-HRPs and work–life conflict. Given the COR theoretical perspective through which the hypotheses were developed, it follows that this study supports the notion that older workers’ wellbeing and turnover intention are influenced by resource losses and gains. Therefore, OW-HRPs can foster a healthier work environment with improved older worker retention.

The results indicate the OW-HRPs were associated with lower levels of Age Discrimination, Work–Life Conflict, Psychological Distress and Turnover Intention. Taken together, the research highlights the important role that OW-HRPs can play in improving the quality of work for an ageing workforce, while also strengthening retention prospects.

## 4. Discussion

The findings in this study have answered calls for research examining the role of HRPs in employee wellbeing [29], and, most importantly, applied much needed attention to the role of HRPs in the wellbeing of older workers [45]. The novel focus of the study on understanding the mechanism by which OW-HRPs specifically designed to support older workers can be adversely impacted by negative work environment factors, such as those experienced during the COVID-19 pandemic, builds on earlier research in the older worker wellbeing field [11,45,46]. In addition, it informs other streams of research, such as business strategy, organizational development, and also informs practitioners such as management consultants [47]. In their study of the impact of COVID-19 on the consulting industry, Szeiner et al. [47] found that COVID-19 provided the opportunity for organizations to adapt to the changing work environment; hence, for management consultants the adoption of OW-HRPs to retain older workers contributes to the range of measures they may introduce to improve organizational performance.

### 4.1. Contribution to Theory

This study extends earlier research regarding the impact of HRPs on older worker wellbeing by examining the mediating effect of negative workplace factors on the intended positive influence of OW-HRPs. Previous research reports that work–life conflict is positively associated with the leave intentions of older workers [34,36]; however, the field lacks studies examining the wellbeing outcomes of work–life conflict amongst older workers. Our novel findings that work–life conflict is associated with undesirable wellbeing and turnover intentions amongst older workers; and that work–life conflict mediates the relationship between OW-HRPs, psychological distress and turnover intentions will have implications for, and build on, existing theory. Further, the high-quality work environment engendered as a result of the provision of supportive OW-HRPs will be negatively impacted where workers perceive a negative work environment such as exposure to work–life conflict and age-discrimination [11]. Furthermore, age-discrimination was found to fully mediate the negative relationship between OW-HRPs and work–life conflict, thereby further reducing the influence of these variables on older workers’ wellbeing and psychological distress.

Responding to the need for a focus on organizational support as a primary means to enhance the psychological wellbeing of older workers [10,30,46,48], this study advances understanding about OW-HRPs. In particular, the findings support the notion that OW-HRPs are a potentially potent organizational resource that can positively influence the psychological wellbeing and retention of older workers. Further, this study reinforces the arguments of COR theory emphasizing that the provision of organizational resources is vital in the context of enhancing older worker wellbeing. This is consistent with contemporary research proposing that older workers may experience higher stress levels than younger workers where organizational resources are low [49]. Thus, the findings highlight the importance of OW-HRPs designed to support older workers in nurturing their psychological wellbeing [9,10,18,30].

### 4.2. Implications for Practice

For organizations seeking to retain older workers during times of uncertainty and disruption due to events such as the COVID-19 pandemic, workforce shortages and ageing populations, our findings suggest that OW-HRPs provide a critical organizational resource that older workers can utilize to help offset resource loss when personal resources are low [49] and to reduce associated work demands and stress [27]. In addition, OW-HRPs signal that the organization values and recognizes the contributions of older workers [19] and strives to enhance the environment to fit with older workers’ needs and preferences, resulting in the positive psychological wellbeing of older workers [30]. In responding to the changing work environment brought on by the impact of COVID-19, many management consultants, HR practitioners and strategy consultants view the pandemic as an opportunity [47]. For these practitioners, our findings suggest that OW-HRPs can support the adaptive responses, such as inclusive organizational cultures, and may even help organizations to position themselves as an employer of choice in a time where workforce shortages are increasingly common.

Given the potential for negative workplace factors to reduce the intended positive influence of OW-HRPs on older worker wellbeing and retention, it is beholden on managers to ensure the work environment is free of ageism and age discrimination [10,48]. In this study, age-discrimination fully mediated the HR practices and work–life conflict relationship, suggesting that the potential benefits of OW-HRPs to support older workers can be lost where ageism and/or age-discrimination is present in the work environment. This result speaks to the potential for a policy-practice divide, whereupon effective OW-HRPs (including flexibility) deployed in an environment of exclusion and ageism, will not create the desired effect of work–life balance for workers. Likely, this is because line managers explicitly or implicitly block a worker’s request for OW-HRPs, despite their being written in workplace policies. Initiatives to promote discrimination-free workplaces include training line-managers in unconscious bias [19,48,50] and age awareness programs designed to challenge bias and recognize the capabilities and limitations associated with ageing [17]. Finally, the implementation of OW-HRPs can promote a common understanding amongst managers and employees that older workers are valued and supported within the organization [10].

### 4.3. Limitations

We acknowledge the limitations associated with using panel data and that our sample may not be representative of other populations, globally. However, the strength of our data was its representativeness in terms of industry, occupational and role distributions, according to national workforce statistics for Australia, with a sample of older workers surveyed across a diverse range of Australian workplaces. In addition, the study experienced the traditional limitations of collecting data from a sample at two different points in time, whereby the lower response rate to the second survey reduced the size of the dataset available for analysis. Moreover, the relatively short period between the two data collection points limited our understanding of the influence of OW-HRPs over time.

### 4.4. Future Research

Future research should focus on the potential role for OW-HRPs in supporting the wellbeing of older workers from relatively high-risk sectors for psychological injury, as these are also the industries with the greatest need to safely engage older workers within the workforce. In addition, further research examining the most effective mechanisms for implementing HRPs to support older workers effectively throughout organizations is needed. Pending further research, investigating the training needs and capabilities of line-managers to ensure the inclusive management of older workers is warranted [48], as are effective ways to promote a healthy work environment where older workers feel valued and supported.

## 5. Conclusions

Maintaining and growing workforce participation rates for older workers is essential, particularly as the COVID-19 pandemic stemmed the movement of skilled workers, highlighted global shortages of skilled labour and emphasized the impact of psychological safety climates on the wellbeing of workers globally. The novel focus of our study on understanding the mechanism by which HRPs designed to support older workers can be adversely impacted by negative work environment factors builds on current research, particularly of those researchers working in the mature-aged and older worker wellbeing field. Further, the findings from this study inform organizational approaches that foster work environments conducive to older worker wellbeing so that they retain key, mature aged talent and maintain effectiveness, in the face of substantial labour supply challenges brought on by the COVID-19 pandemic and ageing populations.

## Figures and Tables

**Table 1:** Participant Characteristics.

Characteristic	Proportion of Respondents
Gender *	
Male	41%
Female	59%
Age	
45–54 years	55.7%
55–64 years	32.3%
65 and older	12.0%
Position/Role	
Front-line workers	57.3%
Line-managers or Coordinators	23.0%
Heads of Department/Senior Manager	13.7%
Executives	6.0%
Sector	
Private sector	67.0%
Public sector	25.3%
Not-For-Profits	7.7%
Industry **	
Education and Training	14.7%
Health care and social assistance	11.3%
Retail Trade	8.3%
Finance	8.0%

* No respondent identified outside of binary gender identifiers. ** Remainder comprised of workers from all other sectors (in portions below 6.8%).

**Table 2:** Instrument reliability and validity.

Construct	Composite Reliability	Average Variance Extracted	Maximum Shared Variance	Inter-Construct Correlations				
				1.	2.	3.	4.	5.
OW-HRPs Practices	0.89	0.67	0.15	0.82				
Age Discrimination	0.93	0.62	0.25	−0.39	0.79			
Work–life conflict	0.90	0.70	0.25	−0.26	0.50	0.84		
Psychological distress	0.91	0.63	0.22	−0.26	0.33	0.47	0.79	
Turnover Intention	0.90	0.75	0.22	−0.38	0.34	0.47	0.45	0.87

**Table 3:** Descriptive and correlations between variables.

	Mean	SD	1.	2.	3.	4.	5.
(1) OW-HRPs	1.98	0.84					
(2) Age Discrimination	1.41	0.52	−0.43 ***				
(3) Work–life Conflict	2.63	0.98	−0.28 ***	0.49 ***			
(4) Psychological Distress	1.71	0.75	−0.29 ***	0.35 ***	0.44 ***		
(5) Turnover Intention	2.44	1.13	−0.42 ***	0.36 ***	0.44 ***	0.38 ***	
(6) Age cohort	1.24	0.44	0.05	0.11^	−0.20 ***	−0.17 **	−0.19 ***

N = 300, ^ *p* < 0.1, ** *p* < 0.01, *** *p* < 0.001

**Table 4:** Direct and Indirect Results from Path Analysis.

Path	Direct Effect	Indirect Effect	Evidence of
HR Mature-Age Practices … → Age Discrimination	−0.43 ***	-	Direct Effect
HR Mature-Age Practices … → Work–Life Conflict	−0.08 N.S.	−0.19 ***	Full Mediation
HR Mature-Age Practices … → Psychological Distress	−0.14 *	−0.15 ***	Full Mediation
HR Mature-Age Practices … → Intention to Leave	−0.26 ***	−0.16 ***	Partial Mediation
Age Discrimination → Work–Life Conflict	0.45 ***	-	Direct Effect
Age Discrimination → Psychological Distress	0.13 ^	0.15 ***	Full Mediation
Age Discrimination → Turnover Intention	0.04 N.S.	0.17 ***	Full Mediation
Work–life Conflict → Psychological Distress	0.34 ***	-	Direct Effect
Work–life Conflict → Turnover Intention	0.21 **	0.10 ***	Partial Mediation
Psychological Distress → Turnover Intention	0.30 ***	-	Direct Effect
Model Fit scores	CFA	Improved CFA	Path
CMIN/DF	2.072	1.842	1.774
CFI	0.915	0.934	0.994
TLI	0.909	0.929	0.968
RMSEA	0.060	0.053	0.051

N = 300, ^ *p* < 0.1, * *p* < 0.05, ** *p* < 0.01, *** *p* < 0.001, N.S. = not significant.

## Data Availability

Data available on request due to ethical restrictions.
